# Evaluation of an algorithm for estimating a patient's life threat risk from an ambulance call

**DOI:** 10.1186/1471-227X-9-21

**Published:** 2009-10-21

**Authors:** Kenji Ohshige, Chihiro Kawakami, Shunsaku Mizushima, Yoshihiro Moriwaki, Noriyuki Suzuki

**Affiliations:** 1 Department of Public Health, Yokohama City University School of Medicine, 3-9 Fukuura, Kanazawa-ku, Yokohama, Japan; 2 Critical Care and Emergency Center, Yokohama City University Medical Center, 4-57 Urafune-cho, Minami-ku, Yokohama, Japan

## Abstract

**Background:**

Utilizing a computer algorithm, information from calls to an ambulance service was used to calculate the risk of patients being in a life-threatening condition (life threat risk), at the time of the call. If the estimated life threat risk was higher than 10%, the probability that a patient faced a risk of dying was recognized as very high and categorized as category A+. The present study aimed to review the accuracy of the algorithm.

**Methods:**

Data collected for six months from the Yokohama new emergency system was used. In the system, emergency call workers interviewed ambulance callers to obtain information necessary to assess triage, which included consciousness level, breathing status, walking ability, position, and complexion. An emergency patient's life threat risk was then estimated by a computer algorithm applying logistic models. This study compared the estimated life threat risk occurring at the time of the emergency call to the patients' state or severity of condition, i.e. death confirmed at the scene by ambulance crews, resulted in death at emergency departments, life-threatening condition with occurrence of cardiac and/or pulmonary arrest (CPA), life-threatening condition without CPA, serious but not life-threatening condition, moderate condition, and mild condition. The sensitivity, specificity, predictive values, and likelihood ratios of the algorithm for categorizing A+ were calculated.

**Results:**

The number of emergency dispatches over the six months was 73,992. Triage assessment was conducted for 68,692 of these calls. The study targets account for 88.8% of patients who were involved in triage calls. There were 2,349 cases where the patient had died or had suffered CPA. The sensitivity, specificity, positive predictive value, negative predictive value, positive likelihood ratio and negative likelihood ratio of the algorithm at predicting cases that would result in a death or CPA were 80.2% (95% confidence interval: 78.6% - 81.8%), 96.0% (95.8% - 96.1%), 42.6% (41.1% - 44.0%), 99.2% (99.2% - 99.3%), 19.9 (18.8 - 21.1), and 0.21 (0.19 - 0.22), respectively.

**Conclusion:**

A patient's life threat risk was quantitatively assessed at the moment of the emergency call with a moderate level of accuracy.

## Background

The demand for emergency medical services is increasing in industrialized countries [1-5]. In many countries, ambulance responses are tailored to give priority to true emergency calls and thus save the lives of patients suffering from serious conditions. A reliable prehospital emergency medical service system is essential in the modern community; however, overuse of an ambulance system can negatively affect its ability to save the lives of critically ill patients.

Demand for ambulance services in Japan has risen rapidly over the last decade [6]. The increased demand for ambulance services has gradually lengthened the time it takes for an ambulance crew to respond and arrive at the scene. As delayed response time reduces the number of patients who survive from sudden cardiac arrest [7-9], priority dispatch of ambulances to patients in a critical condition has become a matter of importance for the Japanese prehospital emergency medical services system.

On October 1st, 2008, the city of Yokohama, Japan started a new emergency medical service system that was designed to dispatch ample emergency medical service staff to patients in a critical condition. Distinguishing patients in a critical condition from patients with non-critical conditions at the moment of the emergency call is known as call triage. The algorithm for call triage used in the new ambulance dispatch system is not a simple flowchart but a mathematical model with coefficients and the constant. The probability of patient being in a life-threatening condition (life threat risk) is calculated with a computerized dispatch system, in which information provided in the call to the emergency service is used to estimate the risk. The present study was conducted to review the algorithm.

## Methods

### Organization of Emergency Medical Services in Japan

In Japan, local governments provide prehospital emergency medical services as a public service. Anyone can use an ambulance free of charge by phoning 119. Most local governments staff ambulances with emergency life-saving technicians who are trained for cardiopulmonary resuscitation and pass a national examination. They play an important role as a first responder in the pre-hospital emergency field. They are allowed to defibrillate, to perform tracheal intubation and to administer a resuscitative drug, epinephrine, during out-of-hospital cardiopulmonary resuscitation under online direction from an emergency medicine physician [10,11]. All patients who received advance life support at the scene were transported to hospitals. The Fire and Disaster Management Agency, Ministry of General Affaires of Japan reported that the national average call-response interval of ambulances was 7.0 minutes in 2007.

### Study population and setting

The data used in the study was collected during the Yokohama New Emergency System from October 1st, 2008 to March 31st, 2009. Yokohama is Japan's second largest city (population 3.58 million, Census 2005). Yokohama's prehospital emergency medical service is unified and managed by the Emergency Medical Division of the Yokohama Safety Management Bureau. The number of ambulances dispatched in 2008 was 146,145, and this number had increased constantly up to 2005, after which the rate of increase reduced [12].

In the system, emergency call workers conduct dispatch. They are the official workers of the Emergency Medical Division who must undergo 140 hours of training in interview technique and dispatch procedure, and are required to pass the city examination. The call workers systematically asked ambulance callers for information: age and sex of the patient(s), and the chief complaint of the patient, and whether they could observe if the caller was not a patient himself/herself. Call workers also interviewed callers to obtain information that was used to assess the patient's life threat risk such as consciousness level, breathing status, walking ability, position (lying down or not), and complexion (face colour and sweating). Age was stratified into six groups. Consciousness level was graded to clear, not clear, unconscious, or unconfirmed. Breathing status was graded to normal, abnormal/dypnea, apnea, or unconfirmed. Walking ability was graded to as usual, walk with support, unable to walk, or unconfirmed. This information was entered into a computer-based triage form during the phone call. The triage form categorized patients into A (there is a probability that the patient faced an imminent risk of dying), B (there is a possibility that the patient faced a risk of dying), or C (the probability that the patient faced a risk of dying was very low) [13], based on previous data [14]. The triage form also quantitatively estimated the patient's life threat risk. If the estimated life threat risk was higher than 10%, the triage form categorized patients into A+ (the probability that the patient faced a risk of dying was very high), and an ambulance, a fast response car, and a fire engine were dispatched. At least one emergency life-saving technician was present in the ambulance and fast response car. If patients are categorized in groups A or B, an ambulance and a fast response car are dispatched. If patients are categorized into C, an ambulance is dispatched [14]. Cases where patients were transported from hospital to hospital were excluded from the call triage target. Cases where the caller did not observe the patient, i.e., they were mere messengers, were judged as inappropriate to be triaged.

### Classifying severity

Ambulance crews judge obvious death as having occurred if bodies show *livor mortis*, cadaveric stiffing, or putrefaction, and therefore do not transport them. If an obvious sign of death is not identified, advanced life support is provided to every patient cardiac and/or pulmonary arrest (CPA) has occurred. Information for patients identified with CPA at the scene was entered into the record system by ambulance crews. The condition of patients when they arrive at an emergency department (ED) by ambulance is routinely logged into the city's computer-based ambulance transport record system. The severity of a patient's condition is usually categorized into one of five levels by physicians at the ED: mild (patient's condition is not serious and the patient does not require hospital admission), moderate (patient's condition is not serious but the patient requires hospital admission), serious (but not life-threatening), life-threatening, or resulted-in-death. In the present study, patient condition was classified into seven categories in order to compare the estimated life threat risk to the patients' state or severity: death confirmed at the scene (they were not transported to hospital), resulted in death at emergency departments, life-threatening condition with CPA, life-threatening condition without CPA, serious but not life-threatening condition, moderate condition, and mild condition.

The data used in this study did not include personal information such as the patients' names and addresses. Use of data from the city's computer-based record system was in accordance with two municipal ordinances enacted by the Yokohama municipal assembly: the Free Access to Information Ordinance (enacted February 25, 2000); and the Protection of Personal Information Ordinance (enacted February 25, 2000). The study was approved by the ethics committee of the Yokohama City University School of Medicine.

### Algorithm for estimating a patient's life threat risk

A computer algorithm estimates a patient's life threat risk. The algorithm was constructed with a logistic model [15]. The probability, P, of the life threat risk as assessed from an emergency call was expressed as:



where β reflects the impact of information x obtained via interview with the caller; 'x' consists of information regarding the patient's consciousness level, breathing status, walking ability, position (standing, sitting, or lying) and other signs such as cyanosis and sweating. Coefficient β differs by the type of caller: a family member, nursing home staff, or third party (not patients themselves, nor family members, nor nursing home staff). If the value of P was higher than 0.1 (10%), patients were categorized as A+.

The values of the coefficients used in the logistic models in the computer algorithm are shown in Table 1. The coefficients of variables were estimated from a trial (sample size was 4,301) prior to the start of the new system with multivariate logistic analyses, in which the independent variables equals 1 if the patient's condition resulted in death or was recognized as life-threatening at the ED, and 0 if classified under one of the less serious categories [14]. In the analyses, age strata, consciousness level, breathing status, and walking ability were treated as categorical variables and other variables were treated as dummy variables. No model exists to estimate the life threat risk from calls made by patients themselves. The algorithm had been used under the Yokohama New Emergency System, which started from October 1st, 2008.

**Table 1:** Coefficients of variables in the logistic model applied for estimating the patient's life threat risk

	**Variables**	**Family members**	**Nursing home staff**	Third party*
Age	0-4	0.000	0.000	0.000
	5-14	0.000	0.000	0.000
	15-39	0.000	0.000	0.000
	40-69	2.231	0.000	1.482
	70+	2.564	1.106	1.807
	Unknown	2.564	1.106	1.807
				
Consciousness	Clear	0.000	0.000	0.000
	Not clear	0.190	1.275	0.822
	Unconsciousness	1.749	2.450	2.568
	Unconfirmed	1.749	0.978	2.568
				
Breathing status	Normal	0.000	0.000	0.000
	Abnormal/dypnea	0.989	18.198	2.132
	Apnea	4.861	20.748	5.654
	Unconfirmed	1.116	17.650	1.541
				
Walking ability	As usual	0.000	0.000	0.000
	Walk with support	0.999	0.000	0.000
	Unable to walk	1.727	15.075	0.086
	Unconfirmed	1.805	16.221	0.000
				
Lying down	Yes	0.536	0.508	0.503
	No	0.000	0.000	0.000
				
Cyanosis	Yes	0.112		
	No	0.000	0.000	0.000
				
Sweating	Unconfirmed	0.724		
	Yes or No	0.000	0.000	0.000
				
Caller is in a panic	Yes	0.501		
	No	0.000	0.000	0.000
				
Constant		-8.780	-38.338	-7.601

*not family members nor nursing home staff

### Review of the algorithm for estimating a patient's life threat risk

First, the patient's estimated life threat risk at the moment of the emergency call was compared with the state or severity of the patient's condition. Second, sensitivity, specificity, predictive values, and likelihood ratios of the cut-off criterion for categorizing patients into A+ were calculated with the 95% confidence interval (95%CI) from the data collected under the new system.

## Results

### Patients

**Figure 1:**
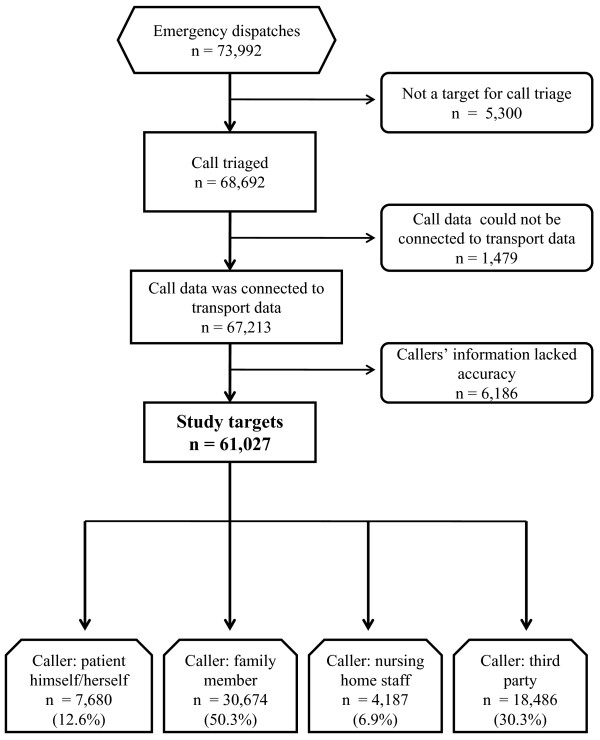
**Flow diagram of the study**. There were 73,992 emergency dispatches for the study period, October 1^st^, 2008 to March 31^st^, 2009 in Yokohama, Japan. The study targets account for 88.8% of patients who were the subject of call triage.

There were 73,992 emergency dispatches from October 1st, 2008 to March 31st, 2009. Call triage was conducted for 68,692 cases out of the emergency dispatches. Out of 68,692 cases, there were 1,479 cases where data obtained from the call triage system could not be connected to the patient's data recorded in the ambulance transport record system because more than one person was transported following the emergency call. Out of the 67,213 cases, 6,186 cases were judged as inappropriate for triage under the triage system because callers were mere messengers and their information lacked accuracy. Consequently, this study was conducted for the remaining 61,027 cases (Figure 1).

Out of the 61,027 cases, 714 were confirmed as dead at the scene and were not transported by ambulance; 538 were transported and confirmed as dead on arrival at the EDs; and 1,803 were recognized as being in a life-threatening condition at the EDs. Ambulance crews reported that 537 (99.8%) of the 538 cases that resulted in death at EDs, and 1,097 (60.8%) of the 1,803 life-threatened cases had been classed as CPA at the scene. There were 3,450 cases, 18,064 cases, and 31,616 cases that were judged at the EDs as serious but in a non life-threatening condition, moderate condition, and mild condition, respectively. Cases that were not transported to EDs because of other reasons such as the patient's refusal or a false call, numbered 4,831.

### Callers

For the 61,027 cases that were the targets of this study, 7,680 calls were made by the patients themselves; among the calls made by patients, 11 cases (0.14%) resulted in death or CPA. The number of calls from family members was 30,674; among those, 1.475 cases (4.8%) resulted in death or CPA. There were 4,187 calls made by nursing home staff; among those, 302 cases (7.2%) resulted in death or CPA. There were 18,486 calls made by third party callers, i.e., people other than the former three types of callers, such as a friend, a passer-by, a police officer or a station attendant; among those, 561 cases (3.0%) resulted in death or CPA.

### Patient's severity and estimated life threat risk

**Figure 2:**
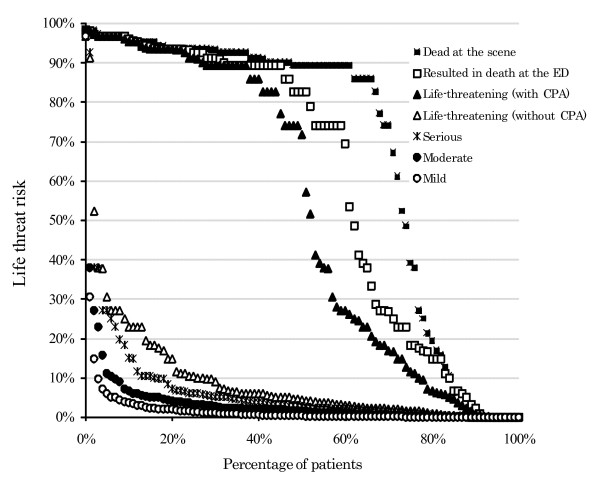
**Life threat risk estimated from emergency calls according to the severity of the patients' condition**. The vertical axis displays the percentage likelihood of each patient facing a life threat risk. The horizontal axis defines the percentage of patients categorized into each condition which are defined as: death confirmed at the scene, resulted in death at emergency departments (EDs), life-threatening condition with occurrence of cardiac and/or pulmonary arrest (CPA), life-threatening condition without CPA, serious condition but not life-threatening, moderate condition, and mild condition. Each defined point in the figure represents one patient transported by ambulance. For instance, this figure shows that patients whose life threat risk is higher than 10% account for approximately 80% of the patients whose condition resulted in death at the ED.

The life threat risk as estimated from the quantitative analyses is shown in Figure 2 by the status of the patient, i.e., death confirmed at the scene, resulted in death at EDs, life-threatening condition at EDs with CPA at the scene, life-threatening condition at EDs without CPA, serious but not life-threatening condition at EDs, moderate condition at EDs, and mild condition at EDs.

Out of the 61,027 cases that were the target of this study, there were 4,423 cases that were categorized as A+. The 4,423 cases included 597 out of 714 cases (83.6%) where death was confirmed at the scene, 447 out of 538 cases (83.1%) that resulted in death at EDs, 839 out of 1,097 cases (76.5%) that resulted in a life-threatening condition at EDs and confirmed CPA at the scene, 180 out of 706 cases (25.5%) that resulted in a life-threatening condition at EDs without CPA, 518 out of 3,450 cases (15.0%) that were classed as having a serious condition but not life-threatening at EDs, 1,026 out of 18,064 cases (5.7%) that were classed as having a moderate condition at EDs, 652 out of 31,616 (2.1%) that were classed as having a mild condition at EDs, and 164 out of 4842 (3.4%) that were not transported to EDs (Table 2). In the cases that were categorized as A+, 23.6% of them were represented by cases where death was confirmed at the scene or resulted in death as defined by physicians at the EDs, and 19.0% of them represented patients classed as having a life-threatening condition with CPA. Thus, the sensitivity, specificity, positive predictive value, and negative predictive value of the categorizing patients as A+ that resulted in death or CPA were 80.2% (95% CI: 78.6% - 81.8%), 96.0% (95% CI: 95.8% - 96.1%), 42.6% (95% CI: 41.1% - 44.0%), and 99.2% (95% CI: 99.2% - 99.3%), respectively. The positive likelihood ratio and negative likelihood ratio of the categorizing were 19.9 (95% CI: 18.8 - 21.1), and 0.21 (95% CI: 0.19 - 0.22), respectively.

**Table 2:** The number and percentage of cases categorized into A+ by state or severity

	**Cases**	Cases categorized A+*	
	**number**	**number**	**%**
Death confirmed at the scene and not transported	714	597	(83.6)
Resulted in death at emergency departments	538	447	(83.1)
Life-threatening condition with CPA	1097	839	(76.5)
Life-threatening condition without CPA	706	180	(25.5)
Serious but not life-threatening condition	3450	518	(15.0)
Moderate condition	18064	1026	(5.7)
Mild condition	31616	652	(2.1)
Other condition	11	0	(0.0)
Not transported because of patient's refusal or a false call	4831	164	(3.4)
Total triage cases conducted	61027	4423	(7.2)

* category A+: estimated life threat risk is higher than 10% at the moment of emergency call

### Accuracy of the triage algorithm by the type of caller

Accuracy of the algorithms for categorizing patients as A+ that resulted in death or CPA differed by the type of caller. Sensitivity, specificity, positive predictive value, negative predictive value, positive likelihood ratio, and negative likelihood ratio of the algorithm for calls from family members were 84.8% (95% CI: 83.0% - 86.7%), 95.7% (95% CI: 95.4% - 95.9%), 49.7% (95% CI: 47.8% - 51.7%), 99.2% (95%CI: 99.1% - 99.3%), 19.6 (95%CI: 18.2 - 21.1), and 0.16 (95%CI: 0.14 - 0.18), respectively. These values of the algorithm for calls from nursing home staff were 91.4% (95% CI: 88.2% - 94.6%), 80.2% (95% CI: 78.9% - 81.4%), 26.4% (95% CI: 23.7% - 29.0%), 99.2% (95%CI: 98.9% - 99.5%), 4.61 (95%CI: 4.2 - 5.1), and 0.11 (95%CI: 0.07 - 0.15), respectively. The values of the algorithm for calls from third party callers were 63.5% (95% CI: 59.5% - 67.4%), 97.2% (95% CI: 97.0% - 97.4%), 41.4% (95% CI: 38.2% - 44.7%), 98.8% (95%CI: 98.7% - 99.0%), 22.6 (95%CI: 19.5 - 26.3), and 0.38 (95%CI: 0.33 - 0.42), respectively.

## Discussion

Because a quick response to emergency calls for patients in a life-threatening conditions is important to improve their survival rate [16], several countries have introduced a priority dispatch system for ambulances [17-24]. On October 1st, 2008, Yokohama, Japan started a new emergency medical service system that was designed to dispatch ample emergency staff quickly to patients in a critical condition. The present study aimed to assess the algorithm, which had originally been constructed based on data collected previously from 4,301 cases, prior to the start of the new system [14].

In the new Yokohama system, when the life threat risk as estimated by the logistic model is higher than 10%, the emergency call is categorized as A+. Category A+ targets patients that face a strong possibility of dying. The Emergency Medical Division of the Yokohama Safety Management Bureau reported that under the new emergency system, the mean arrival time of the first responder to the scene for patients categorized as A+ at the moment of the emergency call was approximately one minute shorter than that for other patients. Whether the new system improved survival rate from CPA should be evaluated in further studies.

Logistic models were applied to construct an algorithm to assess the life threat risk from the information received in calls to emergency services. The algorithm for assessing the life threat risk was constructed according to the type of caller. This is based on a concept that the weight of data obtained from the calls is likely to differ depending on the type of caller. For instance, a call reporting that a patient cannot walk could have different implications when made by nursing home staff compared to other types of callers. The life threat risk was estimated synthetically, from observable signs provided by callers to the ambulance system. The model allowed explanatory variables to be recorded as unknown or unconfirmed. This is based on a concept that information that is unknown or unconfirmed is potentially related to the severity of patient's condition and can be used as a factor in the risk assessment.

In the triage program, the patients' life threat risk was expressed as a percentage. For example, when a call was made by a family member who was in panic, if the patient's age was 70 years, consciousness not clear and breathing status abnormal, if the patient was lying down and unable to walk, the patients face cyanotic, and sweating unable to be confirmed, then the life threat risk was estimated to be 19.2% by the model. In the Yokohama New Emergency System, patients were categorized as potentially life threatened when the estimated life threat risk was higher than 10%. The cut-off value was determined prior to the start of the system according to the city's capability of dispatching ample staff, i.e., from a viewpoint on the amount of acceptable false positives, such as overtriage. In the present algorithm for assessing patients' life threat risk, the term overtriage represents the amount of patients without CPA who were categorized as A+ (the overtriage rate was 57.4%), while undertriage represents the amount of patients with CPA who were not categorized as A+ (the undertriage rate was 0.8%). A high rate of overtriage will result in an inappropriate high priority dispatch from the limited number of ambulances, while a high rate of undertriage will result in an unnecessary loss of lives.

The cut-off value was set as the same value regardless of the type of caller. With the cut-off value, the algorithm for calls made from nursing home staff achieved high level sensitivity (91.4%), meanwhile the sensitivity of the algorithm for calls made from third party was relatively low (63.5%). Sensitivity and specificity have a trade-off relationship. An appropriate cut-off value of the algorithms must be reconsidered.

We included the obviously dead patients in our review because these patients were not identifiable at the time of emergency call. Ambulance crews were dispatched to rescue every patient, among whom persons identified as obviously dead at the scene were included. If obvious death is identified at the scene, patients are not transported to hospitals. When non-transported cases are excluded from the evaluation study, sensitivity, specificity, predictive values, and likelihood ratios are changed. In this case, the sensitivity, specificity, positive predictive value, negative predictive value, positive likelihood ratio, and negative likelihood ratio of categorizing patients as A+ that resulted in death or CPA was 78.7% (95%CI: 76.7% - 80.6%), 95.6% (95%CI: 95.4% - 95.8%), 35.1% (95%CI: 33.6% - 36.7%), 99.3% (95%CI: 99.3% - 99.4%), 17.8 (95%CI: 16.7 - 19.0), and 0.22 (95%CI: 0.20 - 0.24), respectively.

Several studies on the validity of triage systems have been reported in the UK [20,21], Canada[23], Finland [24], USA[25] and Australia [26]. Heward et al. reported that 50% of cardiac arrests were identified by the Advanced Medical Priority Dispatch System [20]. Flynn et al. reported that sensitivity and specificity of the Medical Priority Dispatch System for detecting cardiac arrest were 76.7% and 99.2% [26]. Direct comparison on the accuracy of triage systems is difficult because relevant terms for estimating the accuracy have not been presented in their entirety in the literature. The likelihood ratio incorporates both the sensitivity and specificity of the algorithm and provides a direct estimation of the accuracy of the triage [27,28].

There are several challenges for developing a more improved triage algorithm. The algorithm to assess a patient's life threat risk can be improved with the data obtained under the new emergency medical services system, in which information obtained during emergency calls is recorded as digital data. Although the coefficients of explanatory variables shown in Table 1 were estimated from limited sample data, data of more than 120,000 triaged cases per year should contribute to the development of an accurate triage algorithm. The variables used as explanatory variables in the logistic model were derived from data sought from callers by call workers, for instance, 'how is his consciousness?' Under an emergency situation, the number of such questions is inevitably limited. Patient's age, consciousness level, breathing status, walking ability, position, and complexion were selected as data that a call worker should seek in the interview protocol. There may be factors for assessing the life threat risk other than the variables used in the current algorithm. If other indicative factors are found in the future, they should be part of the interview protocol and should be included as explanatory variables in the model.

The coefficients of the logistic model were estimated by logistic regression analyses whose dependent variable is 1 if the patient's condition resulted in death or was recognized as life-threatening by physicians at the ED. Otherwise the dependent variable was 0. Although the current algorithm was constructed with the dependent variable of such outcome, i.e., 1 or 0 mentioned above, there may be other outcomes or indices that serve as the optimum yardstick for determining advanced life support intervention.

Obtaining accurate information from the initial call to the emergency services is crucial for developing a well-organized algorithm. The information on the patient's condition is quite accurately recorded under the new system because the information was entered into a computer-based triage form during the phone call. In the meantime, the information obtained from callers is prone to being inaccurate if the callers do not observe patients sufficiently to give the accurate information required. Such cases should be excluded from the targets of call triage.

A logistic model does not yet exist that can assess the patient's risk of death when calls are made to emergency services by the patients themselves. Such a model is unlikely to be developed no matter how much data will be collected, because only a small percentage of such cases resulted in a critical condition. Methods other than a quantitative approach may be preferable to predict the chance of a critical condition occurring when an emergency call is made by the patient.

## Conclusion

A patient's life threat risk can be quantitatively expressed at the moment of the emergency call with a moderate level of accuracy. The algorithm for estimating a patient's life threat risk should be improved further as more data are collected.

## Competing interests

The copyright of the computer-based triage form used in the study belongs to Yokohama City University.

## Authors' contributions

KO designed the study and drafted the manuscript. NS and YM managed data collection. KO and CK analyzed the data. SM helped to draft the manuscript. All authors contributed substantially to the revision of the draft manuscript. All authors read and approved the final manuscript.

## Authors' information

KO and NS are members of the Medical Control Council, Yokohama, Japan. NS is also the chairman of the council.

## Pre-publication history

The pre-publication history for this paper can be accessed here:



## References

[B1] Wrigley H, George S, Smith H, Snooks H, Glasper A, Thomas E (2002). Trends in demand for emergency ambulance services in Wiltshire over nine years: observational study. BMJ.

[B2] Nawar EW, Niska RW, Xu J (2007). National Hospital Ambulatory Medical Care Survey: 2005 emergency department summary. Adv Data.

[B3] Lambe S, Washington DL, Fink A, Herbst K, Liu H, Fosse JS, Asch SM (2002). Trends in the use and capacity of California's emergency departments, 1990-1999. Ann Emerg Med.

[B4] Benger JR, Jones V (2008). Why are we here? A study of patient actions prior to emergency hospital admission. Emerg Med J.

[B5] Yang NP, Lee YH, Lin CH, Chung YC, Chen WJ, Chou P (2009). Utilization of and direct expenditure for emergency medical care in Taiwan: a population-based descriptive study. J Epidemiol.

[B6] Kawakami C, Ohshige K, Kubota K, Tochikubo O (2007). Influence of socioeconomic factors on medically unnecessary ambulance calls. BMC Health Services Research.

[B7] Larsen MP, Eisenburg MS, Cummins RO, Hallstrom AP (1993). Predicting survival from out-of-hospital cardiac arrest: a graphic model. Ann Emerg Med.

[B8] Pell JP, Sirel JM, Marsden AK, Ford I, Cobbe SM (2001). Effect of reducing ambulance response times on deaths from out of hospital cardiac arrest: cohort study. BMJ.

[B9] Moore W, Kee F, Evans AE, McCrum-Gardner EE, Morrison C, Tunstall-Pedoe H (2005). Pre-hospital coronary care and coronary fatality in the Belfast and Glasgow MONICA populations. Int J Epidemiol.

[B10] Ohshige K, Shimazaki S, Hirasawa H, Nakamura M, Kin H, Fujii C, Okuchi K, Yamamoto Y, Akashi K, Takeda J, Hanyuda T, Tochikubo O (2005). Evaluation of out-of-hospital cardiopulmonary resuscitation with resuscitative drugs: a prospective comparative study in Japan. Resuscitation.

[B11] Tanigawa K, Tanaka K (2006). Emergency medical service systems in Japan: past, present, and future. Resuscitation.

[B12] Ohshige K (2008). Reduction in ambulance transports during a public awareness campaign for appropriate ambulance use. Acad Emerg Med.

[B13] Harvey L, Woollard M (2004). Outcome of patients identified as dead (beyond resuscitation) at the point of the emergency call. Emerg Med J.

[B14] Ohshige K (2007). Triage algorithm in Yokohama dispatch system. Kyu-kyu Iryo Journal.

[B15] Greene WH (2003). Models for discrete choice. Econometric Analysis Upper Saddle River.

[B16] Steg PG, Bonnefoy E, Chabaud S, Lapostolle F, Dubien PY, Cristofini P, Leizorovicz A, Touboul P (2003). Impact of time to treatment on mortality after prehospital fibrinolysis or primary angioplasty: data from the CAPTIM randomized clinical trial. Circulation.

[B17] Schmidt TA, Cone DC, Mann NC (2004). Criteria currently used to evaluate dispatch triage systems: where do they leave us?. Prehosp Emerg Care.

[B18] Shah MN, Bishop P, Lerner EB, Fairbanks RJ, Davis EA (2005). Validation of using EMS dispatch codes to identify low-acuity patients. Prehosp Emerg Care.

[B19] Marks PJ, Daniel TD, Afolabi O, Spiers G, Nguyen-Van-Tam JS (2002). Emergency (999) calls to the ambulance service that do not result in the patient being transported to hospital: an epidemiological study. Emerg Med J.

[B20] Heward A, Damiani M, Hartley-Sharpe C (2004). Does the use of the Advanced Medical Priority Dispatch System affect cardiac arrest detection?. Emerg Med J.

[B21] Deakin CD, Sherwood DM, Smith A, Cassidy M (2006). Does telephone triage of emergency (999) calls using Advanced Medical Priority Dispatch (AMPDS) with Department of Health (DH) call prioritisation effectively identify patients with an acute coronary syndrome? An audit of 42,657 emergency calls to Hampshire Ambulance Service NHS Trust. Emerg Med J.

[B22] Clawson J, Olola CH, Heward A, Scott G, Patterson B (2007). Accuracy of emergency medical dispatchers' subjective ability to identify when higher dispatch levels are warranted over a Medical Priority Dispatch System automated protocol's recommended coding based on paramedic outcome data. Emerg Med J.

[B23] Feldman MJ, Verbeek PR, Lyons DG, Chad SJ, Craig AM, Schwartz B (2006). Comparison of the medical priority dispatch system to an out-of-hospital patient acuity score. Acad Emerg Med.

[B24] Kuisma M, Holmström P, Repo J, Määttä T, Nousila-Wiik M, Boyd J (2004). Prehospital mortality in an EMS system using medical priority dispatching: a community based cohort study. Resuscitation.

[B25] Shah MN, Bishop P, Lerner EB, Czapranski T, Davis EA (2003). Derivation of emergency medical services dispatch codes associated with low-acuity patients. Prehosp Emerg Care.

[B26] Flynn J, Archer F, Morgans A (2006). Sensitivity and specificity of the medical priority dispatch system in detecting cardiac arrest emergency calls in Melbourne. Prehosp Disaster Med.

[B27] Deeks JJ, Altman DG (2004). Diagnostic tests 4: likelihood ratios. BMJ.

[B28] Kahn CA, Schultz CH, Miller KT, Anderson CL (2009). Does START triage work? An outcomes assessment after a disaster. Ann Emerg Med.

